# Role of c-miR-21, c-miR-126, Redox Status, and Inflammatory Conditions as Potential Predictors of Vascular Damage in T2DM Patients

**DOI:** 10.3390/antiox11091675

**Published:** 2022-08-27

**Authors:** Gabriela C. López-Armas, Arailym Yessenbekova, Rocío E. González-Castañeda, Kevin J. Arellano-Arteaga, Ana Guerra-Librero, Nurzhanyat Ablaikhanova, Javier Florido, Germaine Escames, Darío Acuña-Castroviejo, Iryna Rusanova

**Affiliations:** 1 Departamento de Investigación y Extensión, Centro de Enseñanza Técnica Industrial, C. Nueva Escocia 1885, Guadalajara 44638, Mexico; 2 Department of Biophysics, Biomedicine and Neuroscience, Al-Farabi Kazakh National University, Al-Farabi Av. 71, Almaty 050040, Kazakhstan; 3 Laboratorio de Microscopia de Alta Resolución, Departamento de Neurociencias, Centro Universitario de Ciencias de la Salud (CUCS), Universidad de Guadalajara, Sierra Mojada 950, Guadalajara 44340, Mexico; 4 División de Medicina Interna, Nuevo Hospital Civil Juan I. Menchaca, Universidad de Guadalajara, Salvador Quevedo y Subieta 750, Guadalajara 44340, Mexico; 5 Centro de Investigación Biomédica en Red Fragilidad y Envejecimiento Saludable (CIBERFES), Instituto de Investigación Biosanitaria de Granada (Ibs), 18016 Granada, Spain; 6 Centro de Investigación Biomédica, Instituto de Biotecnología, Parque Tecnológico de Ciencias de la Salud, Universidad de Granada, 18016 Granada, Spain; 7 Department of Physiology, Faculty of Medicine, University of Granada, 18016 Granada, Spain; 8 Department of Biochemistry and Molecular Biology I, Faculty of Science, University of Granada, 18019 Granada, Spain

**Keywords:** type 2 diabetes mellitus, vascular complications, circulating miR-21, miR-126, IL-6, oxidative stress markers

## Abstract

The development of type 2 diabetes mellitus (T2DM) vascular complications (VCs) is associated with oxidative stress and chronic inflammation and can result in endothelial dysfunctions. Circulating microRNAs play an important role in epigenetic regulation of the etiology of T2DM. We studied 30 healthy volunteers, 26 T2DM patients with no complications, and 26 T2DM patients with VCs, to look for new biomarkers indicating a risk of developing VCs in T2DM patients. Peripheral blood samples were used to determine redox state, by measuring the endogenous antioxidant defense system (superoxide dismutase, SOD; catalase, CAT; glutathione reductase, GRd; glutathione peroxidase, GPx; and glucose-6-phosphate dehydrogenase, G6DP) and markers of oxidative damage (advanced oxidation protein products, AOPP; lipid peroxidation, LPO). Additionally, inflammatory marker levels (IL-1, IL-6, IL-18, and TNF-α), c-miR-21, and c-miR-126 expression were analyzed. T2DM patients showed the highest oxidative damage with increased GSSG/GSH ratios, LPO, and AOPP levels. In both diabetic groups, we found that diminished SOD activity was accompanied by increased CAT and decreased GRd and G6PD activities. Diabetic patients presented with increased relative expression of c-miR-21 and decreased relative expression of c-miR-126. Overall, c-miR-21, SOD, CAT, and IL-6 had high predictive values for diabetes diagnoses. Finally, our data demonstrated that IL-6 exhibited predictive value for VC development in the studied population. Moreover, c-miR-21 and c-miR-126, along with GPx and AOPP levels, should be considered possible markers for VC development in future studies.

## 1. Introduction

Type 2 diabetes mellitus (T2DM) is a complex metabolic disease characterized by hyperglycemic states resulting from tissue insulin resistance [1]. It is predicted that by 2030, up to 10.2% of the world’s population between 20 and 79 years of age will suffer from T2DM [1]. The appearance of T2DM typically occurs 4–7 years before diagnosis; prolonged endothelial cell exposure to chronic hyperglycemia leads to endothelial dysfunction, which, is considered one of the leading causes of T2DM complications [2]. Cardiovascular disease is the major complication related to diabetes, and around 75% of T2DM patients die due to cardiovascular damage, including coronary artery disease, stroke, and peripheral arterial disease [1]. T2DM patients also have an increased risk for microvascular complications, including retinopathy, neuropathy, and nephropathy. Clinical studies show that treatments aiming only at glycemic control are ineffective in reducing vascular complications [3]. There is a need for new therapeutic targets to mitigate vascular injury and prevent development of vascular complications in people with diabetes.

The pathophysiology of vascular complications associated with diabetes is complex and multifactorial. Hyperglycemia in non-insulin-dependent cells (e.g., endothelial cells) leads to metabolic changes, mitochondrial dysfunction, increased oxidative stress (OS), chronic inflammation, and epigenetic modifications by microRNA [4].

Increased oxidative metabolism in the cell contributes to the highest electron escape in the electron transport chain, increased redox potential, and generation of the superoxide radical (O2^−•^) [5]. Moreover, intracellular hyperglycemia leads to glucose auto-oxidation and the formation of advanced glycation products (AGEs) that contribute to the production of the hydroxyl radical (HO^•^) [5]. The increase in reactive oxidative species (ROS), in general, and in O2^−•^, in particular, generates an increase in protein kinase C (PKC) activity, activating oxidative stress signals (through NADH oxidase) and activating inflammation by the NF-κB pathway and NLRP3 inflammasome (“the nucleotide-binding and oligomerization domain, leucine-rich repeat, and pyrin domain-containing 3”) [6]. The depletion of the cofactor NADPH affects the functioning of glutathione reductase and nitric oxide synthase (eNOS), contributing to increasing levels of reactive oxygen and nitrogen species (ROS and RNS, respectively) and worsening vasodilatation. The lowest antioxidant defense in diabetics contributes to OS too. However, there is a scarcity of data regarding full redox status and oxidative stress markers in T2DM, and its relation to human vascular complications [7,8].

MicroRNAs (miRNAs) are involved in various pathological processes, including diabetic vascular complications [9,10]. miRNAs are small, non-coding endogenous RNAs, with approximately 20–25 nucleotides, that are able to modulate specific mRNA expression by pairing to the 3′untranslated region (3′UTR), causing inhibition of gene expression at the post-transcriptional level [11]. miRNAs have been detected and extracted from different biological fluids, including whole blood, serum, and plasma; so, they are very stable molecules and easily detectable in circulation [12]. Changes in circulating miRNA (c-miRNA) expression can help predict, diagnose, and monitor metabolic diseases, including diabetes [12,13,14].

The main objective of this study was to determine how markers of oxidative stress and other inflammatory parameters change in people with diabetes, with and without complications. At the same time, we analyzed the relative expression of two circulating miRNAs: miR-21, related to chronic inflammation, and miR-126, associated with protective endothelial function. For this, we used blood samples from healthy people and patients with T2DM, either with VCs or without complications, from Guadalajara, Mexico. Mexico is the country with the highest prevalence of diabetes among those belonging to the Organization for Economic Cooperation and Development (OECD), reaching 13.8% of their population [15]. Identifying and using biomarkers involved in the pathological progression of disease allow for more accurate identification of disease processes. This knowledge will enable the search for new treatments to prevent vascular complications in T2DM.

## 2. Materials and Methods

### 2.1. Participants and Setting

The New Civil Hospital Juan I. Menchaca Ethical Committee approved this study by Clinical Assays, Mexico (ref no: 17 CI 14 039 116 COFEPRIS), and by the Ethics Committee of the University of Granada, Spain (ref no: 940/CEIH/2019). All participants gave informed consent before laboratory selection. The study was carried out with a total of 82 participants, classified into the three following groups:

Control (CG): Healthy volunteers with normal response to glucose and insulin, without a family history of first-degree diabetes, who did not have any endocrine, vascular, cardiac, or inflammatory pathologies, and was not taking any concomitant medication.

T2DM NC: Patients with controlled type 2 diabetes mellitus, without vascular complications and normal creatinine levels.

T2DM + C: Patients with type 2 diabetes mellitus that had micro- (diabetic retinopathy, nephropathy, and neuropathy) or macrovascular complications (stroke, coronary artery disease, and peripheral arterial insufficiency). All vascular events were documented in the patient’s clinical file or clinical assessment.

The general criteria for inclusion for all groups comprised participants being over 40 years old and diagnosed with T2DM at least five years prior. Exclusion criteria included the existence of cancer, dialysis, amputations, pregnancy, inflammatory, infectious, or autoimmune diseases, and having a BMI greater than 40 kg/m^2^. The diagnosis of patients with T2DM was based on the criteria of the American Diabetes Association (ADA): fasting plasma glucose ≥ 126 mg/dL (7.0 mM), oral glucose tolerance test ≥ 200 mg/dL (11.1 mM), or glycosylated hemoglobin (HbA1c) ≥ 6.5%/48 mM [16]. Anthropometric parameters (height, weight, and waist circumference), medical history, and blood pressure were collected at enrollment of study or during medical visits.

Peripheral blood from the participants was collected from the antecubital veins in tubes containing EDTA after a 12-h overnight fast. Samples were centrifuged at 3500 r/min for 15 min at 4 °C. Plasma and erythrocytes were aliquoted in RNase/DNase-free tubes and stored at −80 °C until further testing.

### 2.2. Biochemical Analysis

Biochemical parameters were analyzed in the clinical analysis service area of the Nuevo Hospital Civil Juan I. Menchaca, Guadalajara. Measurements of total cholesterol (TC) and triglyceride (TG) levels were assayed by colorimetric enzymatic methods. Fasting plasma glucose (FPG) was detected by the Spein method using a Siemens analyzer (Siemens Healthcare, Erlangen, Germany), glycosylated hemoglobin (HbA1c) was measured by an automated analyzer (Tosoh, Japan), and insulin levels were detected by a Centaur XP automatic biochemical analyzer (Siemens, Siemens Healthcare, Erlangen, Germany). The homeostasis model assessment for insulin resistance (HOMA-IR) was calculated using the equation: [fasting plasma glucose (mg/dL) × fasting insulin (uU/mL)/405]. Renal status was assessed by measuring the serum concentration of creatinine as an indicator of glomerular filtration rate and urea as an indicator of impaired excretory function of the kidney.

### 2.3. Circulating miRNAs Profiling and Data Analysis

Plasma miRNAs were isolated using the miRNAeasy Serum/Plasma Advanced kit (Qiagen, Werfen España, ref. 217204), following the manufacturer’s instructions. Reverse transcription and qPCR for selected miRNAs were performed using the TaqMan MicroRNA Advanced kit (Thermo Fisher Scientific, Life Technologies, Thermo Fisher Scientific, Madrid, Spain) and TaqMan Advanced miRNA assays for each miRNA (miR-21-5p: assay 477975_mir; miR-126-5p: assay 477888_mir; miR-143-3p: assay 477912_mir) (Thermo Fisher Scientific), following the manufacturer’s protocol.

Mature extracted miRNAs (2 μL) were modified by extending the 3′ end by poly(A) addition, then the 5′ end was lengthened using an adaptor ligation reaction in a final volume of 15 μL. Finally, the modified miRNAs were reverse transcribed in a final volume of 30 μL, and 5 μL of the RT reaction products were amplified by a miR-Amp reaction obtaining a uniform pool of cDNA. Real-time PCR was performed in a final volume of 20 μL, using a mix of TaqMan Fast Advanced Master Mix with miRNA TaqMan Advanced Assays specific for our analysis, through the Agilent Technologies Stratagene Mx3005P System (Agilent Technologies, Spain). All real-time PCR reactions were performed in triplicate. The threshold cycle values were determined using a fixed threshold setting, and the average ≥32 Ct were not included in the analysis. Data were analyzed using the SDS 2.3 and RQ Manager 1.2 software packages (Life Technologies), and relative gene expression was calculated using the 2−∆∆CT method (∆Ct target miR-∆Ct control gene). miR-143 was used as the internal control and miR-39 as the external control. The expression of miRNA of each patient was calculated against the Ct of the control group on the plate.

### 2.4. Measurement of Plasma Cytokines

HCYTA-60K-03 Human Cyto Panel A for IL-6, IL-10, IL-18, and TNF-α (Milliplex) was used to analyze the profile expression of cytokines in the plasma fraction, following the manufacturer’s instructions. A Luminex 200 system (Luminax xMAP technology, Thermo Fisher Scientific, Madrid, Spain) was used to analyze each cytokine based on the corresponding standard curve. The concentrations were determined using xPonent 3.1 software (Luminex Corporation, Austin, TX, USA) and were expressed in pg/mL.

### 2.5. Measurement of AOPP and LPO Levels

Advanced oxidation protein products (AOPP) and products of lipid peroxidation (LPO) were determined in plasma samples. As previously described, AOPPs were measured spectrophotometrically on a microplate reader [17]. The standard curve was made with a chloramine-T solution in the presence of potassium iodide (0–100 nmol/mL) and 20 μL of acetic acid, measuring absorbance at 340 nm. The absorbance of the reaction mixture was determined on a microplate reader against a blank containing 200 μL PBS, 10 μL potassium iodide, and 20 μL of acetic acid. The AOPP concentration was expressed in nmol/mL of chloramine-T equivalents. LPO was determined by a commercial colorimetric kit (KB03002, Bioquochem kit, BQC Redox Technologies, Asturias, Spain) that estimates both malondialdehyde (MDA) and 4-hydroxyalkenals. All procedures were done according to the manufacturer’s instructions. Absorbance was read at 586 nm, and LPO concentration was expressed in nmol/mL [18].

### 2.6. Measurement of GSSG and GSH Levels

Glutathione disulfide (GSSG) and reduced glutathione (GSH) levels were measured in the erythrocyte fraction by a fluorimetric method using a microplate fluorescence reader (FLx800; BioTek Instruments Inc., Highland Park, VT, USA) [19]. Results were expressed as μmol/g Hb.

### 2.7. Measurement of CAT, GPx, GRd, G6PD, and SOD Activities

Catalase (CAT), glutathione peroxidase (GPx), glutathione reductase (GRd), glucose-6-phosphate dehydrogenase (G6PD), and superoxide dismutase (SOD) activities were determined in the erythrocyte fraction. According to Aebi’s method, CAT activity was measured following the decomposition of H_2_O_2_ at 240 nm [20]. GPx and GRd activities were spectrophotometrically measured following NADPH oxidation for 3 min at 340 nm in a 96-well plate spectrophotometer (PowerWaveX; BioTek) [21]. GRd activity was measured using a kit (703202; Cayman chemical, Ann Arbor, MI, USA), following the manufacturer’s instructions. Erythrocyte G6PD activity was determined by measuring the rate of change in absorbance at 340 nm, due to the reduction of NADP^+^, using the G-6PD kit (Spinreact, Cromakit, S.L., Granada, Spain). Enzyme activity was expressed as μmol/min/g Hb. SOD activity was assessed in terms of its ability to inhibit the auto-oxidation of adrenalin to adrenochrome at a pH of 10.2. Oxidation of adrenalin was measured at 490 nm for 10 min at 30 °C as described in [22]. SOD activity was expressed as U/mg Hb (1 unit = 50% inhibition of auto-oxidation of epinephrine).

### 2.8. Statistical Analysis

Data were analyzed using SPSS version 25.0 (WPSS Ltd., Surrey, UK), and graphs were generated using GraphPad Prism v. 6.0 for Windows scientific software (GraphPad Software Inc., La Jolla, CA, USA). Continuous data were tested for normality using the Shapiro-Wilk test, and Levene´s statistic verified the homogeneity of variance. Analysis of covariance (ANCOVA) followed by multiple-comparison post-hoc tests were used to compare the mean differences in continuous variables between groups. After adjusting for age and sex, a one factor ANOVA with a Kruskal-Wallis test was used for non-normally distributed variables. A Pearson´s test was performed to determine correlations between quantitative variables. Receiver operating characteristic (ROC) curves were constructed to evaluate the diagnostic value of miRNAs and other markers. The area under the curve (AUC) and 95% confidence intervals (CI) were calculated to determine specificity and sensitivity. Binomial logistic regression analysis was used to explore the association between plasma miRNAs, oxidative/inflammatory biomarker levels, and the presence of diabetes/complications. Results are displayed as odds ratios (ExpB) and 95% confidence intervals (CIs). Differences were considered statistically significant at *p* values < 0.05.

## 3. Results

### 3.1. Characteristics of the Studied Subjects

The study included 82 participants of all sexes, divided into three groups: control (*n* = 30, 14 women and 16 men), T2DM patients without complications (*n* = 26, 7 women and 19 men), and T2DM with complications (*n* = 26, 14 women and 12 men). All groups had BMIs greater than 25, which put them in the overweight or obese categories. However, there were no significant differences between groups, reflecting the widespread problem of obesity in the Mexican population. Patients with complications presented with significantly higher systolic pressure, which is to be expected in this type of patient.

Table 1 shows the profiles and biochemical parameters of the study participants. Among the biochemical parameters, we observed significantly higher HbA1c, glucose, and HOMA-IR index levels in both diabetic groups compared with the control group. Total cholesterol and triglyceride levels were not significantly different among the groups.

Table 2 summarizes the distribution of micro- (retinopathy, nephropathy, and neuropathy) and macro- (stroke, heart failure, and peripheral arterial disease) vascular complications in the T2DM + C group. Different applied therapies are also indicated.

### 3.2. Plasma miRNA Expression in Studied Samples

**Figure 1:**
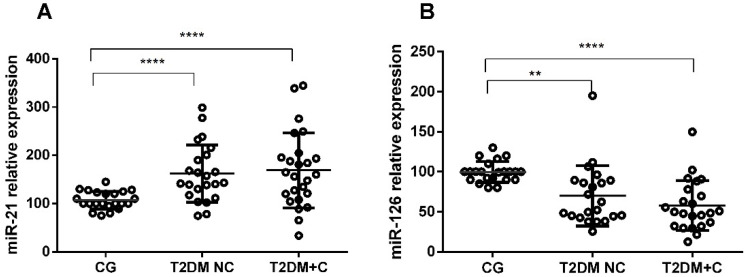
Relative expression of circulating (**A**) miR-21-5p and (**B**) miR-126-5p in T2DM patients without complications (T2DM NC), diabetics with complications (T2DM + C), and in controls (CG). Data are presented as means ± standard error of the mean (SEM). Comparisons between groups are indicated in the graphs. ** *p* < 0.01, and **** *p* < 0.0001.

**Figure 2:**
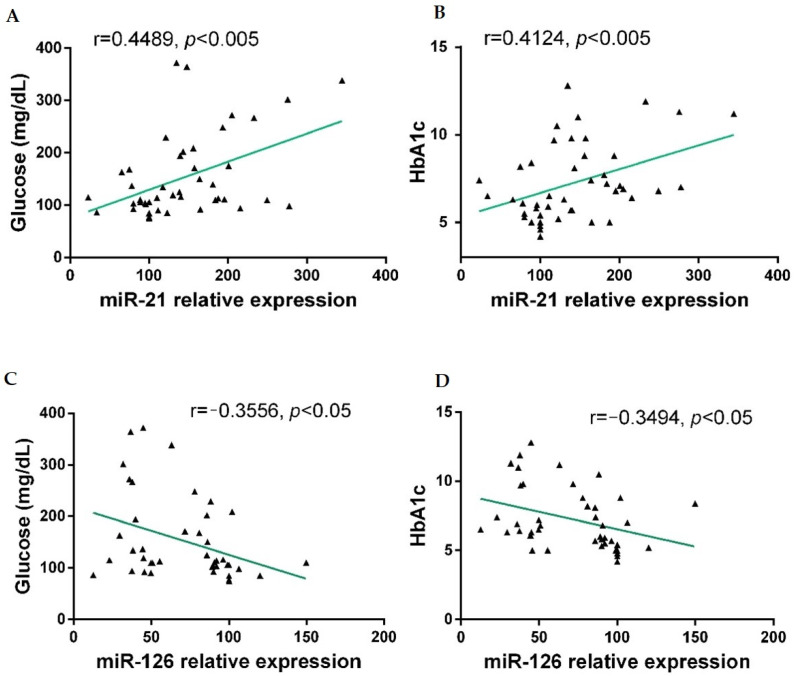
Correlations of relative circulating miR-21 and miR-126 with biochemical parameters in participants were calculated using Pearson correlation coefficient (r) analysis. Correlations between relative miR-21 expression and glucose levels (**A**), miR-21 and HbA1c (**B**), relative miR-126 expression and glucose (**C**), and miR-126 and HbA1c (**D**) were significative.

The real-time PCR results showed that the plasma level of miR-21 was significantly higher in diabetic groups with and without complications compared with the control group (*p* < 0.001). On the contrary, levels of miR-126 expression decreased markedly in the T2DM NC group (*p* < 0.01) and in T2DM + C patients compared with the healthy group (*p* < 0.001) (Figure 1A,B). The Pearson correlation analysis revealed positive correlations between miR-21 expression and glucose, as well as between miR-21 and HbA1c levels (*p* < 0.005, Figure 2A,B). In contrast, miR-126 levels were negatively correlated with both parameters (*p* < 0.05) (Figure 2C,D).

### 3.3. Oxidative Status and Markers of Inflammation in Studied Samples

**Figure 3:**
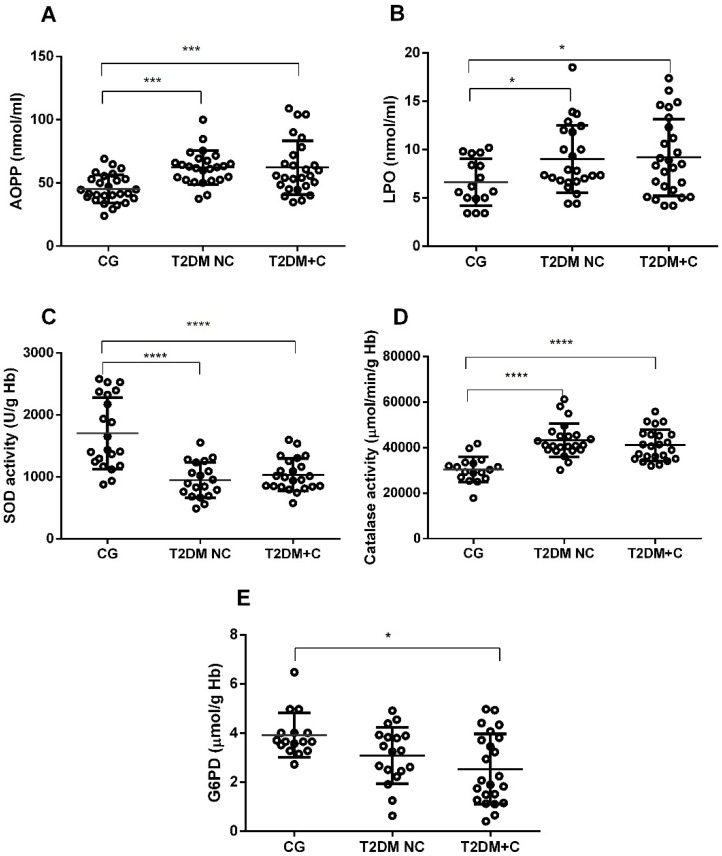
Analysis of oxidative stress parameters in human plasma and erythrocytes of diabetic patients without complications (T2DM NC), with vascular complications (T2DM + C), and controls (CG). (**A**) Advanced oxidation protein products (AOPP) in plasma, (**B**) plasma lipid peroxidation (LPO), (**C**) erythrocyte superoxide dismutase activity (SOD), (**D**) catalase (CAT), and (**E**) glucose-6-phosphate dehydrogenase (G6PD) activity were measured. Comparisons between groups are indicated in the graphs. Data are presented as mean ± SEM. * *p*< 0.05, *** *p*< 0.001 and **** *p*< 0.0001.

Determination of extracellular oxidative status through analysis of LPO and AOPP levels in plasma revealed that T2DM NC and T2DM + C patients presented significantly higher levels of both markers, with *p* < 0.001 for AOPP and *p* < 0.05 for LPO (Figure 3A,B).

The study of intracellular oxidative status included measuring antioxidant enzyme activity (CAT, SOD, and G6PD) and glutathione cycle components (GSSG, GSH, GRd, and GPx) in erythrocytes. Concerning antioxidant enzymes, we detected decreased SOD activity (*p* < 0.001, Figure 3C) and increased CAT activity (*p* < 0.001, Figure 3D) in both diabetic groups compared with the control group. There was lower G6PD activity in the T2DM + C group than in controls (*p* < 0.05, Figure 3E).

**Figure 4:**
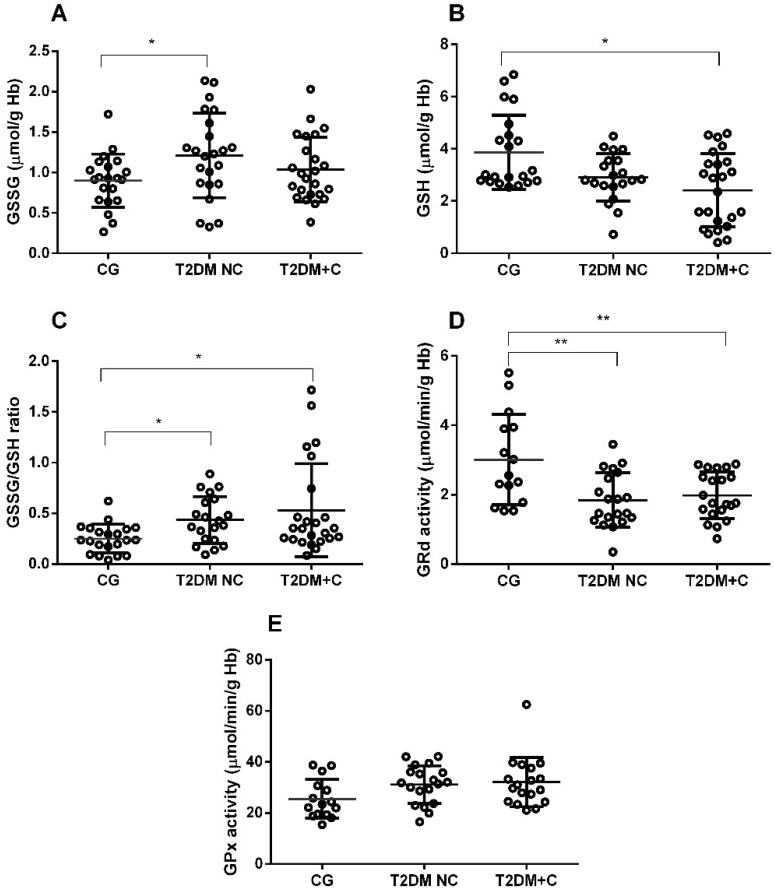
Glutathione cycle parameters were measured in the erythrocytes from CG (control group), T2DM NC (diabetics without complications), and T2DM + C (ones with complications). The following parameters were measured: (**A**) *p* levels of oxidized glutathione (GSSG), (**B**) reduced glutathione (GSH), (**C**) GSSG/GSH ratio, (**D**) glutathione reductase (GRd), and (**E**) glutathione peroxidase (GPx) activity. Comparisons between groups are indicated in the graphs. Data are presented as mean ± SEM. * *p* < 0.05, ** *p* < 0.01.

Regarding glutathione cycle components, GSSG levels were significantly higher in the T2DM NC group and significantly lower GSH in the T2DM + C group, compared with healthy controls (*p* < 0.05; Figure 4A,B). Additionally, we found an increased GSSG/GSH ratio in both diabetes groups compared with healthy participants. (*p* < 0.05; Figure 4C). GRd presented significantly lower activity in both diabetes groups compared with the control group (*p* < 0.01); however, GPx had no significant changes in activity (Figure 4D,E).

**Figure 5:**
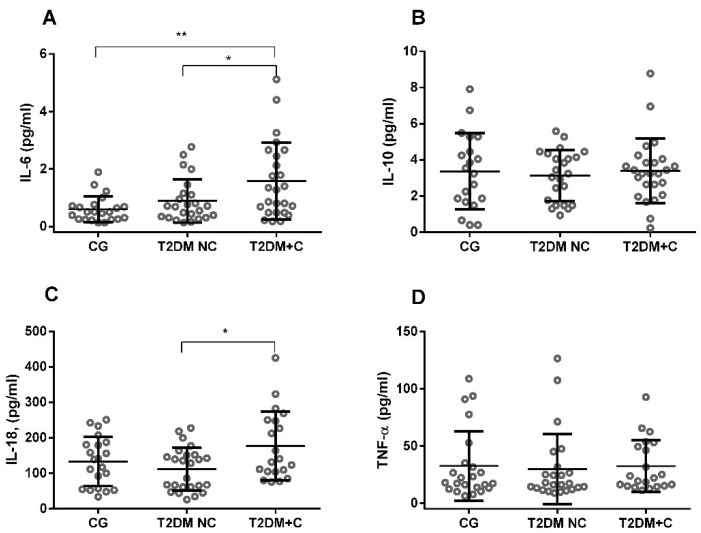
The concentration of pro- (IL-6, IL-18, and TNF-α) and anti-inflammatory cytokines (IL-10) in plasma samples from diabetic and control groups. (**A**) IL-6; (**B**) IL-10; (**C**) IL-18; and (**D**) TNF-α were assessed. Comparisons between groups are indicated in the graphs. Data are presented as mean ± SEM. * *p* < 0.05, ** *p* < 0.01, vs. control.

We also analyzed four inflammatory cytokines in plasma from T2DM patients and controls. The levels of IL-6 have significantly increased in T2DM + C groups compared with controls (*p* < 0.01, Figure 5A), and the highest levels of IL-6 were detected in the T2DM + C group compared with the T2DM NC group. Moreover, IL-18 was significantly increased in the T2DM + C group compared with the T2DM NC group (*p* < 0.05, Figure 5C). No significant differences, however, were observed in IL-10 and TNF-α levels.

Regarding the glutathione cycle, a significant Pearson correlation analysis revealed significance between some parameters when comparing oxidative stress markers and biochemical variables in all participants (Table 3). It is worth highlighting the significant positive correlations between AOPP and analytical parameters (glucose levels, HbA1c, and TG), such as between CAT and HbA1c, CAT activity and the HOMA-IR index, GSSG/GSH ratio and TG, and LPO and IL-6.

In the same way, negative correlations were found between GRd and glucose, GRd and HbA1c, SOD and glucose, SOD and HbA1c, and SOD and TG. Moreover, negative correlations were found between G6PD and analytical parameters (HbA1c, TG, and urea) (see Table 3). * *p* < 0.05, ** *p* < 0.01, *** *p* < 0.001, and **** *p* < 0.0001.

Some of these correlations lost their significance when analyzed only in the diabetic groups. It is worth noting that the correlation between GPx activity and glucose levels was close to being significant (r = 0.359, *p* = 0.06). However, there were significant correlations between: GPx and IL-10 (r = −0.358, *p* = 0.037); GPx and TNF-α/IL-10 (r = 0.461, *p* = 0.008); and AOPP and TNF-α/IL-10 (r = 0.393, *p* = 0.009). In addition, there was an approximating correlation between years since diagnosis and G6PD.

Also, we analyzed the correlations between the expressions of miRNAs and oxidative and inflammatory marker status in all participants and in the diabetic group only, adjusting for age, sex, and BMI in both cases (Table 4). All participants had a negative correlation between miR-21 and SOD, and a positive correlation between miR-21 and GPx. The latter was maintained in diabetic groups. In addition, significant negative correlations between miR-21 and IL-10, and miR-126 and IL-6 were found in diabetic patients, adjusting for sex, age, and BMI. The positive correlations between miR-126 and GPx, and miR-126 and the GSSG/GSH ratio were found in diabetic patients; the latter was maintained in diabetic patients, adjusting for sex, age, and BMI (r = 0.493, *p* = 0.017).

### 3.4. Diagnostic Accuracy of Study Biomarkers for Diabetes and Vascular Complications

To study the diagnostic accuracy of circulating miR-21 and miR-126, as well as oxidative stress and inflammatory markers, receiving operator characteristic (ROC) curves were drawn. In Table 5, we observe AUC and 95% confidence intervals for analyzed markers in all participants. It should be noted that several markers appear as candidates for having a significant predictive value for diabetes. We selected only biomarkers with sensitivity > 50% and AUC > 750, as follows: HbA1c, CAT, miR-21, GPx, AOPP, and LPO (these markers are underlined in Table 5).

However, when ROC analysis was completed within the T2DM NC and T2DM + C groups to assess the predictive power of markers for developing vascular complications, these differences disappeared, except for GPx and IL-6 (Table 6).

Logistic regression models compared classical diagnostic parameters for diabetes diagnosis with biochemical and plasmatic biomarkers. Notably, some of these models revealed the highest predictive risk for diabetes:

miR-21, SOD, and IL-6: Exp (B) = 2.235, chi2 = 49.015, *p* = 0.006;

miR-21, SOD, IL-6, and LPO: Exp (B) = 4.22, chi2 = 50.731, *p* = 0.001; and

miR-21, CAT, IL-6, and BMI: Exp (B) = 3.818, chi2 = 46.107, *p* = 0.001.

**Figure 6:**
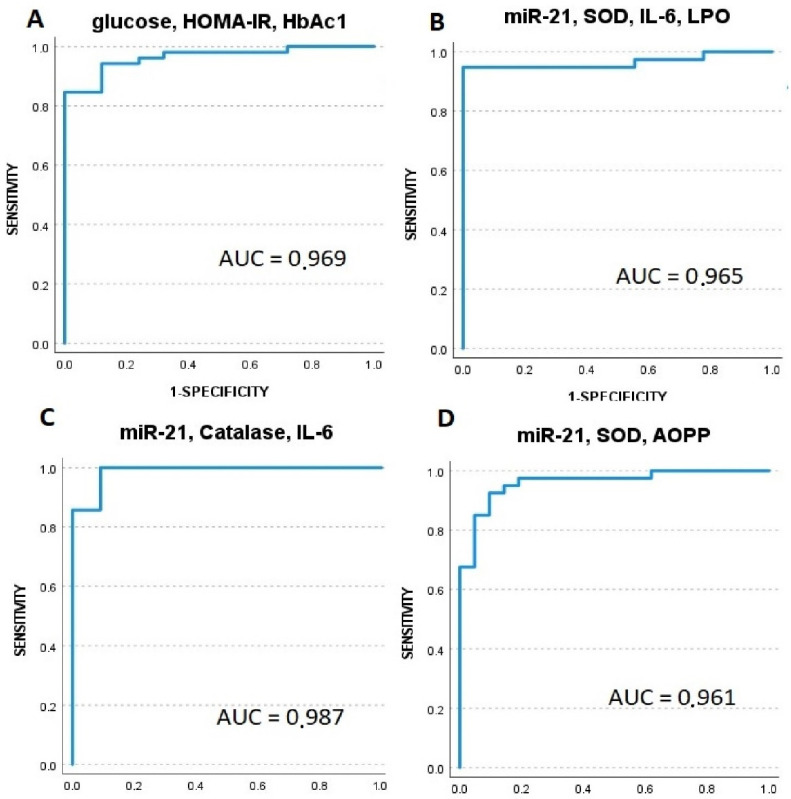
Receiver operator characteristic (ROC) curves generated for sensitivity analysis show diagnostic performances of plasma markers including the following models: (**A**)-glucose, HOMA-IR, HbA1c; (**B**)-miR-21, SOD, IL-6, LPO; (**C**)-miR-21, catalase, IL-6; (**D**)-miR-21, SOD, and AOPP. AUC-area under the curve.

We applied ROC curve analysis to evaluate the predictive values of these markers. Results demonstrated that AUC values for models with miR-21, SOD, IL-6, and LPO and miR-21, SOD, and AOPP were similar (Figure 6B,D) or higher (model with miR-21, CAT, and IL-6) than the predictive AUC value for classical diagnostic parameters (glucose, HbA1c, and HOMA-IR) (Figure 6A,C).

To analyze the diagnostic value of the different parameters for predicting the risk of developing complications, we performed binary logistic regression analysis. When we compared other models of classic clinical parameters, such as glucose, HbA1c, years since diagnosis, glucose, and urea, only the following models had a significant value, although with lower specificity: miR-126 and IL-6; miR-126 and years since diagnosis; miR-21 and IL-6; and miR-21, IL-6, AOPP. See Table 7.

We also assessed the diagnostic performance of different models in discriminating the T2DM NC and T2DM + C phenotypes. However, binary logistic regression analysis revealed that only IL-6 had a significant predictive value for the development of vascular complications in the diabetic groups: Exp (B) = 2.347, OR: 1.136–4.848, *p* = 0.021.

## 4. Discussion

Type 2 diabetes mellitus is associated with oxidative stress and low-grade inflammation resulting in endothelial dysfunction (ED). Clinical studies demonstrate that tight glycemic control does not significantly reduce the appearance of diabetic vascular complications [3]. On the other hand, increased oxidative stress and inflammation can lead to insulin resistance and impair insulin secretion. Additionally, changes in epigenetic control can influence these processes [2]. There is a need to analyze changes in inflammatory and oxidative state markers and miRNA expression to find biological markers that have sufficiently high predictive power for the development of vascular complications in diabetic patients.

In this study, we found significantly increased c-miR-21 and decreased c-miR-126 plasma levels in diabetes patients with and without complications, compared with healthy controls, in the Mexican population (Figure 1), even after adjusting for sex and age (data not shown). These results are consistent with several published studies. In humans, increased plasma miR-21 is considered a predictor of later development of T2DM in prediabetic individuals [23]. Studies have reported that miR-21 exerts its deleterious actions in diabetic retinopathy [24], diabetic nephropathy [25], beta-cell apoptosis [26], and is associated with pancreatic islet inflammation and insulin resistance initiation [27]. An in vitro study showed that high levels of glucose increased miR-21 expression [28]. This study revealed a positive correlation between c-miR-21 and glycemic impairment (plasmatic glucose and HbA1c) in diabetic patients. Additionally, miR-21 is considered a pro-inflammatory miRNA induced by several pro-inflammatory molecules [26]; increasing miR-21 leads to promoting the NF-κB pathway and subsequent NLRP3 inflammasome activation, acting directly on Toll-like receptors and by targeting A20 [26,29].

As a result, it is reasonable to expect increased miR-21 to lead to higher production of pro-inflammatory ILs. Functional in vitro experiments with mimic miR-21 elevated the levels of IL-6 and IL-1β and reduced IL-10 [30]. In this study, we did not find a relation between miR-21 expression and pro-inflammatory ILs; nevertheless, we found a negative correlation between miR-21 and anti-inflammatory IL-10 in diabetic patients, after adjusting for sex, age, and BMI (Table 4). Our results are similar to those of a recent publication that showed that miR-21 inhibition in myeloid cells in a sepsis model was associated with increased anti-inflammatory IL-10 in vivo and in vitro [31,32].

Otherwise, c-miR-126 showed lower levels in T2DM NC and T2DM + C than controls. These results are consistent with previous studies that reported reduced c-miR-126 levels as a biomarker for pre-diabetes and T2DM [33,34,35]. Different studies have demonstrated that miR-126 decreases VCAM-1 expression and can promote insulin resistance by inhibiting IRS-1 [36,37]. It has been shown to be involved in regulating vascular integrity and angiogenesis and is one of the most expressed miRNAs in the endothelium [38]. Animal model studies have revealed that reduced expression of miR-126 is significantly associated with T2DM [39,40] and brain damage [39]. On the contrary, high expression of miR-126 is associated with a lower activity of NLRP3, reducing inflammation related to retinopathies [40] and induced brain damage in the mouse diabetic model [39]. The current study also correlated decreased plasma miR-126 with high blood glucose and HbA1c (Figure 2). This correlation suggests that worsened hyperglycemia parameters might result in the reduced delivery of miR-126 to the circulatory system, contributing to endothelial dysfunction.

The notable contribution of this work is a complete study of the redox status in T2DM patients in peripheral blood samples, as well as an analysis of how the antioxidant system responds to oxidative stress in diabetes patients with and without VCs. First, we found that patients, regardless of whether they had complications, had elevated levels of AOPP and LPO compared with controls. AOPP and LPO are products of reactions involving ROS with protein side chains and lipids [40,41,42]. High plasma AOPP concentrations were related to atherosclerotic lesions and vascular inflammation in diabetic patients [40,43]. Moreover, AOPP levels were proposed as an independent risk factor for endothelial dysfunction in individuals in the early stages of diabetes without albuminuria [40]. Recent animal model and in vitro studies demonstrated that AOPPs are not merely OE products but also promoters of redox-sensitive inflammation in the vasculature, mitochondrial dysfunction, and oxidative stress, causing early diabetic nephropathy [42,44]. These findings, in part, were confirmed in our study when we found significant positive associations between AOPP levels and glycemic impairments (plasmatic glucose and HbA1c) and TG. Moreover, the AUC of AOPP exhibited high values in diabetes patients compared with controls (Table 5), which may make this marker a good predictor for diabetes. In addition, the inclusion of this marker in a stepwise logistic regression with miR-21 and IL-6 revealed a significantly high association with the development of vascular complications in diabetic groups (Table 7).

Concerning the antioxidant defense of diabetic patients, we found a significant reduction in erythrocyte SOD and GRd activities and significant increase in CAT activity in diabetic patients, regardless of VCs, compared with controls. In the same way, the T2DM + C group showed decreased G6PD activity versus the control group.

Superoxide dismutase is a primary defense enzyme because it is involved in decomposing toxic superoxide anions to produce H_2_O_2_, which is then converted to non-toxic H_2_O and O_2_ by GPx and CAT. We measured cytoplasmatic SOD, which has Cu/Zn in its active center and is the most sensitive to oxidative stress and high glucose levels. Approximately 50% of the decrease in activity of SOD could be explained by the enzymatic glycosylation of its center of enzymatic activity under hyperglycemic conditions [45]. Based on these data, it is altogether feasible that we found significant negative correlations between SOD and glucose levels, HbA1c, and TG in this study (see Table 3). Reports about SOD activity in diabetes are controversial, as some researchers reported no change in SOD activity, whereas others found an increase [46]. Our results align with those observed in pre-diabetics compared with control [47] and in T2DM patients versus control [48]. Studies in animal diabetic models also detected decreased SOD activity [49].

Catalase is predominantly responsible for catalyzing the conversion of H_2_O_2_ into H_2_O and O_2_ in human erythrocytes [50]. Although a decrease in CAT activity was reported in some studies in patients with nephropathy [51,52]; in others, its increased activity may mean a more significant effort made by the erythrocytes to counteract the accumulation of H_2_O_2,_ as a result of SOD inactivity [8]. A positive correlation between catalase activity and HbA1c, and HOMA (Table 3), should be noted. Thus, the ROC curve analysis suggested that CAT activity changes give it high predictive power for diabetes (Table 5).

The second enzyme in erythrocytes that is responsible for the disposal of H_2_O_2_ is glutathione peroxidase (GPx) [53]. This enzyme is part of the glutathione cycle, using the electrons and protons that GSH releases when converted to GSSG to reduce ROS. GPx responses in T2DM were controversial in different publications. GPx was increased in patients with uncontrolled T2DM compared with pre-diabetics [45,46] and decreased compared with controls [54,55]. With our data in mind, we suggest that GPx activity may increase in conditions of excessive H_2_O_2_ production as an adaptive response against the excess hydrogen peroxide. Gunawardena et al., reported higher GPx activity in T2DM patients with poor glycemic control that was in parallel with the MDA levels [45]. In the present study, GPx activity tended to increase, but this increase was not significant, probably due to a small sample size. Moreover, GPx activity was significantly and positively correlated with TNF-α/IL-10 and increased miR-21 expression, the latter in both diabetic and nondiabetic groups adjusted for sex, age, and BMI (Table 4). Notably, the AUC of GPx exhibited high and significant values not only when at risk for developing diabetes, but also in developing vascular damage in the diabetic group (see Table 5 and Table 6).

A decreased GSH may depend on the reduction of GRd activity or competition for NADPH between GRd and G6PD in the polyol pathway. The latter explanation makes sense because we find that diabetic patients show a positive correlation between GSH and G6PD (r = 0.312, *p* = 0.031). Several patients and animal model studies detected decreased GSH, GRd, and G6PD levels [8,56,57,58]. As a result, an increased GSSG/GSH ratio had a significant positive correlation with TG, and an almost significant positive correlation with glucose, supporting the idea that this oxidative stress marker is related to poor metabolic conditions.

**Figure 7:**
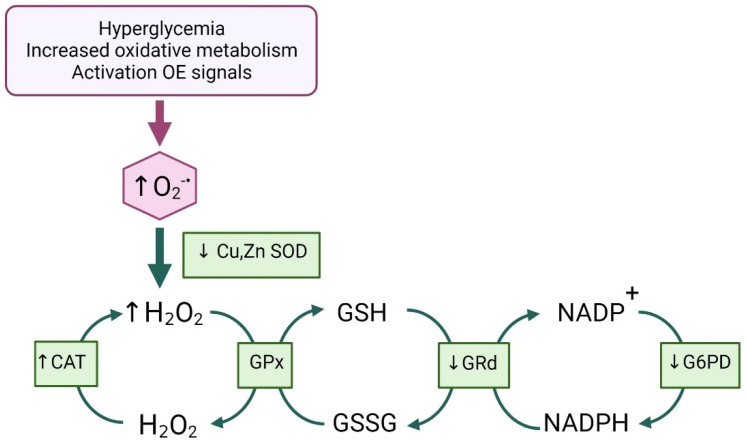
Summary of redox status changes detected in the population studied. Significant reduction of glucose-sensitive SOD activity causes an increase in CAT compensative activity, and GPx activity tends to increase. Decreased G6PD activity limits NADPH levels, contributing to the reduction of GRd activity, which leads to an increased GSSG/GSH ratio. All arrows indicate the directions of biochemical reactions. Created with BioRender.com, (accessed on 16 August 2022).

The exact mechanisms that affect glutathione metabolism in patients with diabetic complications are unclear, and further studies are needed to evaluate the observed problems. However, our study provides complete information on the redox status in the blood of Mexican diabetics (See Figure 7).

This study revealed significantly increased IL-6 and IL-18 levels in the T2DM + C group with respect to the T2DM NC group, as well as compared with the control group in the case of IL-6. Experimental animal studies demonstrated that activation of the NF-κB transcription factor and the NLRP3 inflammasome are involved in a chronic inflammatory response to high oxidative stress in T2DM, which leads to an increase in IL-1β and IL-18, playing a crucial role in the development of diabetes [58], aggravating pro-coagulant activity [6]. Recent studies correlated the overexpression of pro-inflammatory (IL-1β, IL-6, TNF-α, and VEGF) and profibrotic genes (ICAM-1 and VCAM-1) with the appearance of nephropathies in diabetic patients [59]. In our study, IL-6 showed high predictive value for VC development, demonstrated using ROC curves and binary logistic regression analysis. However, IL-6 is highly associated with overweightness and obesity, both states that are related to chronic low-grade inflammation. In this study, the three groups of participants had no significant differences in BMI, weight, or waist circumference, which makes us think that the increase in IL-6 is due not only to weight gain but also to the worsening inflammatory state caused by the disease. Binary logistic regression analysis revealed that models including IL-6 and miR-21, and AOPP had significant predictive value for the development of VCs in the studied population.

Furthermore, although we did not find differences in IL-10 levels between groups, a significant negative correlation between miR-21 and IL-10 was detected in diabetic patients after adjusting for sex, age, and BMI (Table 4). These findings are consistent with several authors who have reported that disequilibrium between pro-inflammatory and anti-inflammatory cytokines aggravates the complications related to T2DM [8,60]. Studies in animal models also confirm the active participation of ILs in developing diabetic complications, such as retinopathy [61] or neuropathy [62].

This was the first systematic investigation of the relationship between plasma microRNA expression and a broad spectrum of oxidative stress parameters in the diabetic population. Notably, our correlational analysis showed an inverse link between miR-21 expression and SOD activity in all participants, corroborating LaSala´s results that demonstrated that miR-21 was associated with a prediabetic status and exhibited predictive value for diabetes detection [24,29]. In addition, we found the enzyme CAT to be a candidate for predicting diabetes, with a high sensitivity, specificity, and AUC, similar to HbA1c. As the disease progresses, a positive correlation between miR-21 and GPx makes us think that GPx plays an essential role in evaluating the risk of suffering complications. This positive correlation is maintained even in the analysis of diabetic patients only, adjusted for sex, age, and BMI (Table 4).

We also corroborated the regulatory role of c-miRNAs in regulating the inflammatory state; in this case, increased c-miR-21 expression was correlated with decreased production of anti-inflammatory IL-10, and decreased miR-126 levels were negatively correlated with pro-inflammatory IL-6. Both correlations were detected in diabetic patient groups after adjusting for sex, age, and BMI (Table 4).

Using both ROC curve analysis and stepwise logistic regression analysis, we identified that models including miR-21, IL-6, CAT, and SOD had a high predictive value for T2DM diagnosis, similar to classical diagnostic parameters (Figure 6). Therefore, we suggest that IL-6 be considered a predictor for the development of vascular complications in the diabetic population. Additional studies are needed to evaluate the possible predictive power of miR-21, miR-126, GPx, and AOPP for the development of vascular complications. In addition, LPO and SOD markers should also be considered for future studies.

A limitation of this study was the reduced sample size. Additionally, the T2DM NC group included a different number of men and women than the other two groups due to randomized recruitment, although there were no significant differences in studied parameters between sexes.

## 5. Conclusions

In the studied diabetic Mexican population, we found increased relative expression of c-miR-21 and decreased relative expression of c-miR-126 in patients versus controls. miR-21, SOD, CAT, and IL-6 revealed a high predictive value for diabetes diagnosis. We suggest considering LPO and AOPP levels as predictive markers for T2DM in future studies. A significant contribution of this study was the complete analysis of redox status and response of antioxidant systems in varying conditions of type 2 diabetes. As the disease progresses, increased CAT activity compensates for decreased SOD activity, and GPx activity tends to increase. Decreased GRd and G6PD activity led to an increased GSSG/GSH ratio. Finally, we found that IL-6 has significant predictive value for the development of vascular damage in the diabetic population with high levels of obesity. Overall, our analyses indicated that a combination of increased c-miR-21 and decreased c-miR-126, with elevated AOPP levels and GPx activity, correlated with vascular complications and should be considered for future studies.

## Figures and Tables

**Table 1:** Baseline characteristics and biochemical parameters of different groups of participants included in the study.

Parameters	CG (*n* = 30)	T2DM NC (*n* = 26)	T2DM + C (*n* = 26)	*p*-Value
Age (years)	49.64 ± 1.78	53.54 ± 2.00	55.31 ± 1.91	
Gender (women/men)	14/16	7/19	14/12	
Weight (kg)	77.61 ± 3.57	87.50 ± 3.77	78.12 ± 3.19	
BMI (kg/m^2^)	27.90 ± 0.77	30.57 ± 1.25	29.49 ± 1.28	
Waist circumference (cm)	94.5 ± 3.03	104.96 ± 2.78	103.34 ± 2.21	
Systolic pressure	121.44 ± 2.9	132.69 ± 3.9	139.65 ± 5.1 ^b^	^b^*p* < 0.01
Diastolic pressure	78.1 ± 1.4	85.3 ± 2.1	84.8 ± 2.7	
Years of diabetes	-	7.85 ± 0.76	11.69 ± 1.5	
HbA1c (%)	5.28 ± 0.09	7.68 ± 0.37 ^a^	8.49 ± 0.44 ^b^	^a,b^*p* < 0.001
Glucose (mg/dL)	92.95 ± 2.02	154.87 ± 10.01 ^a^	197.47 ± 17.10 ^b,c^	^a,b^*p* < 0.001 ^c^ *p* < 0.05
Insulin (mU/L)	9.22 ± 0.87	12.77 ± 1.61	10.77 ± 1.37	
HOMA-IR Index	1.71 ± 0.20	4.09 ± 0.66 ^a^	5.16 ± 0.84 ^b^	^a^*p* < 0.05 ^b^ *p* < 0.001
Total cholesterol (mg/dL)	204.75 ± 6.55	194.12 ± 7.24	197.83 ± 9.30	
TG (mg/dL)	144.52 ± 14.83	165.08 ± 15.62	167.88 ± 15.7	

CG: Non. T2DM NC: T2DM without complications. T2DM + C: T2DM with complications. ^a^ *p*—T2DM NC vs. CG in the post-hoc analysis. ^b^ *p*—T2DM + C vs. CG in the post-hoc analysis. ^c^ *p*—T2DM + C vs. T2DM NC in the post-hoc analysis.

**Table 2:** Types of VCs and medications in the T2DM + C group.

Drug Therapy	Retinopathy (*n* = 4)	Nephropathy (*n* = 3)	Neuropathy (*n* = 14)	CVD (*n* = 5)
	15.4%	11.5%	53.8%	19.2%
Glucose-lowering medication: Antihyperglycemic agents *	100.0%	100.0%	92.8%	60.0%
Insulin therapy	50.0%	0.0%	57.1%	80.0%
Antihypertensive drugs	50.0%	66.6%	50.0%	40.0%
Cholesterol-lowering therapy	25.0%	0.0%	21.4%	0.0%
Anticonvulsants	0.0%	0.0%	28.6%	20.0%

* Antihyperglycemic agents include metformin and second-line glucose-lowering medication (DPP-4 inhibitors, SGLT2 inhibitors, GLP-1 receptor agonists, insulin secretagogues, and thiazolidinediones). Some people need insulin therapy with other antihyperglycemic medications. Painful diabetic neuropathy was treated with anticonvulsants. CVD: Cardiovascular disease. DPP-4: dipeptidyl peptidase-4. SGLT2: sodium-glucose cotransporter 2. GLP-1: glucagon-like peptide type 1.

**Table 3:** Summary of significant (*p* < 0.05) Pearson correlations between oxidative stress markers and biochemical variables for all participants.

	Glucose		HbA1c		HOMA-IR		TG		Urea		IL-6	
	r	*p*	r	*p*	r	*p*	r	*p*	r	*p*	r	*p*
GSSG/GSH	0.240	0.054	0.295	0.244	0.244	0.056	*0.338*	*0.006*	0.142	0.258	0.253	0.053
GRd	*−0.348*	*0.008*	*−0.358*	*0.007*	−0.227	0.102	−0.216	0.110	−0.024	0.858	−0.233	0.100
Catalase	0.200	0.115	*0.261*	*0.039*	*0.262*	*0.047*	0.207	0.104	−0.048	0.707	0.074	0.584
SOD	*−0.283*	*0.024*	*−0.396*	*−0.001*	−0.163	0.212	*−0.289*	*0.021*	−0.173	0.175	−0.189	0.158
G6PD	−0.219	0.102	*−0.408*	*0.002*	0.029	0.835	*−0.270*	*0.042*	*−0.261*	*0.050*	*−0.182*	*0.196*
AOPP	*0.283*	*0.012*	*0.236*	*0.038*	0.226	0.053	*0.506*	*0.000*	0.204	0.073	0.093	0.452
LPO	0.174	0.162	0.176	0.157	0.126	0.333	0.031	0.805	0.110	0.378	*0.338*	*0.009*

In italics, statistical significance.

**Table 4:** Summary of significant (*p* < 0.05) Pearson correlations (r) between plasma miRNA levels and oxidative stress and inflammatory measurements in all participants.

miRNA	Oxidative Stress Parameters	r-Value	*p*-Value
**All participants**			
miR-21	SOD	−0.325	0.001
	GPx	0.443	0.001
	AOPP	0.342	0.019
miR-126	SOD	0.282	0.030
	CAT	−0.359	0.007
	LPO	−0.287	0.027
**All participants adjusted for sex, age, and BMI**			
miR-21	GPx	0.558	<0.0001
miR-126	IL-6	−0.480	0.004
**Diabetic patients**			
miR-21	GPx	0.412	0.014
miR-126	GPx	0.360	0.039
	GSSG/GSH	0.320	0.041
**Diabetic patients adjusted for sex, age, and BMI**			
miR-21	GPx	0.419	0.047
	IL-10	−0.453	0.020
miR-126	IL-6	−0.466	0.016
	GSSG/GSH	0.493	0.017

**Table 5:** AUC and 95% confidence intervals for studied markers distinguishing between control and T2DM groups.

	AUC	95% Confidence Interval		*p*-Value
		Min	Max	
HbA1c	0.957	0.902	1.000	0.000
HOMA IR	0.769	0.626	0.911	0.027
Urea	0.587	0.462	0.712	0.194
miR-21	0.877	0.772	0.983	0.000
miR-126	0.245	0.078	0.411	0.034
GSSG/GSH	0.721	0.488	0.953	0.069
AOPP	0.792	0.642	0.941	0.003
LPO	0.775	0.561	0.990	0.023
Catalase	0.913	0.830	1.000	0.000
GRd	0.249	0.104	0.394	0.004
G6PD	0.277	0.147	0.408	0.010
SOD	0.125	0.036	0.214	0.000
GPx	0.796	0.549	0.897	0.023
IL-6	0.778	0.470	0.811	0.150
IL-10	0.487	0.209	0.766	0.917
IL-18	0.597	0.353	0.840	0.426
TNF-A	0.557	0.303	0.811	0.640

**Table 6:** AUC and 95% confidence intervals for studied markers distinguishing between T2DM NC and T2DM + C.

	AUC	95% Confidence Interval		*p*-Value
		Min	Max	
HbA1c	0.633	0.390	0.875	0.281
Urea	0.578	0.374	0.853	0.332
miR-21	0.520	0.355	0.685	0.810
miR-126	0.325	0.133	0.517	0.101
Catalase	0.371	0.131	0.612	0.295
IL-6	0.708	0.490	0.927	0.040
IL-18	0.720	0.507	0.932	0.074
AOPP	0.511	0.261	0.762	0.926
GPx	0.796	0.549	0.897	0.023
G6PD	0.402	0.200	0.604	0.357
SOD	0.650	0.443	0.856	0.161

**Table 7:** This table represents the predictive values for developing vascular diabetic complications among all participants, calculated by binary logistic regression analysis.

	AUC, (95%)	Exp (B) = OR	Chi ^2^	*p*
Glucose, years since Dx, HbA1c	0.860 (0.782–0.937)	0.464	33.192	0.001
Glucose, years since Dx, urea	0.888 (0.820–0.956)	0.464	38.394	0.001
miR-126, IL-6	0.793 (0.673–0.913)	0.579	18.880	0.041
miR-126, years since Dx	0.868 (0.785–0.950)	0.523	30.655	0.012
miR-21, IL-6	0.810 (0.701–0.920)	0.600	17.879	0.048
miR-21, IL-6, AOPP	0.801 (0.687–0.915)	0.600	19.522	0.048

## Data Availability

All data supporting reported results are contained within the article.
